# Acute kidney injury in the perioperative period and in intensive care units (excluding renal replacement therapies)

**DOI:** 10.1186/s13613-016-0145-5

**Published:** 2016-05-27

**Authors:** Carole Ichai, Christophe Vinsonneau, Bertrand Souweine, Fabien Armando, Emmanuel Canet, Christophe Clec’h, Jean-Michel Constantin, Michaël Darmon, Jacques Duranteau, Théophille Gaillot, Arnaud Garnier, Laurent Jacob, Olivier Joannes-Boyau, Laurent Juillard, Didier Journois, Alexandre Lautrette, Laurent Muller, Matthieu Legrand, Nicolas Lerolle, Thomas Rimmelé, Eric Rondeau, Fabienne Tamion, Yannick Walrave, Lionel Velly, Société française d’anesthésie et de réanimation (Sfar), Société de réanimation de langue française (SRLF), Groupe francophone de réanimation et urgences pédiatriques (GFRUP), Société française de néphrologie (SFN)

**Affiliations:** Service de Réanimation Polyvalente, IRCAN (Inserm U1081, CNRS UMR7284 et CHU de Nice, Hôpital Pasteur 2, 30 Voie Romaine, CHU de Nice, 06000 Nice, France; Service de Réanimation, Hôpital Marc Jacquet, 77000 Melun, France; Service de Réanimation Polyvalente, CHU de Nice, 30 Voie Romaine, 06000 Nice, France; Service de Réanimation médicale, CHU de Clermont-Ferrand, 63000 Clermont-Ferrand, France; Service de Réanimation, Assistance Publique-Hôpitaux de Paris, Hôpital Saint-Louis, 1 Avenue Claude Vellefaux, 75010 Paris, France; Service de Réanimation, Assistance Publique-Hôpitaux de Paris, Hôpital d’Avicenne, 125 rue de Stalingrad, 93000 Bobigny, France; Département de Médecine périopératoire, Hôpital Estaing, CHU de Clermont-Ferrand, 1 place Louis Aubrac, 63000 Clermont-Ferrand, France; Service de réanimation, hôpital de la Charité, CHU de Saint-Etienne, 44 rue Pointe Cadet, 42100 Saint-Etienne, France; Département d’anesthésie-réanimation, Assistance Publique-Hôpitaux de Paris, hôpital Kremlin-Bicêtre, 78, rue de la division du général Leclerc, 94270 Le Kremlin-Bicêtre, France; Service de Pédiatrie, hôpital Sud, CHU de Rennes, 16 Bd Bulgarie, 35203 Rennes, France; Service de Pédiatrie, Néphrologie, hôpital des Enfants, CHU de Toulouse, 330 avenue de Grande-Bretagne, 31059 Toulouse Cedex, France; Service d’anesthésie-réanimation, Assistance Publique-Hôpitaux de Paris, hôpital Saint-Louis, 1, Avenue Claude-Vellefaux, 75010 Paris, France; Service d’Anesthésie Réanimation II, Hôpital du Haut-Lévêque, CHU de Bordeaux, 33600 Pessac, France; Service de néphrologie-dialyse, hôpital Édouard-Herriot, Hospices Civils de Lyon, 5, Place d’Arsonval, 69003 Lyon, France; Service de réanimation, Assistance Publique-Hôpitaux de Paris, hôpital Européen Georges Pompidou, 20, rue Leblanc, 75908 Paris, France; Service de réanimation, hôpital Gabriel Montpied, CHU de Clermont-Ferrand, 58 rue Montalemberg, 63003 Clermont-Ferrand, France; Service de réanimation, hôpital Carémeau, CHU de Nîmes, 4 rue du Professeur Robert-Debré, 30029 Nîmes, France; Service d’anesthésie-réanimation, hôpital Saint-Louis, Assistance Publique-Hôpitaux de Paris, 1, Avenue Claude-Vellefaux, 75010 Paris, France; Service de réanimation, centre hospitalier universitaire, CHU d’Angers, 4 rue Larrey, 49100 Angers, France; Service d’anesthésie réanimation, hôpital Édouard-Herriot, Hospices Civils de Lyon, 5, Place d’Arsonval, 69003 Lyon, France; Service de néphrologie, hôpital Tenon, Assistance Publique-Hôpitaux de Paris, 4, rue de la Chine, 75020 Paris, France; Service de réanimation médicale, hôpital Charles-Nicolle, CHU de Rouen, 1 rue de Germont, 76031 Rouen, France; Service d’anesthésie-réanimation, hôpital de la Timone, Assistance Publique-Hôpitaux de Marseille, 13385 Marseille Cedex 5, France

## Abstract

Acute kidney injury (AKI) is a syndrome that has progressed a great deal over the last 20 years. The decrease in urine output and the increase in classical renal biomarkers, such as blood urea nitrogen and serum creatinine, have largely been used as surrogate markers for decreased glomerular filtration rate (GFR), which defines AKI. However, using such markers of GFR as criteria for diagnosing AKI has several limits including the difficult diagnosis of non-organic AKI, also called “functional renal insufficiency” or “pre-renal insufficiency”. This situation is characterized by an oliguria and an increase in creatininemia as a consequence of a reduction in renal blood flow related to systemic haemodynamic abnormalities. In this situation, “renal insufficiency” seems rather inappropriate as kidney function is not impaired. On the contrary, the kidney delivers an appropriate response aiming to recover optimal systemic physiological haemodynamic conditions. Considering the kidney as insufficient is erroneous because this suggests that it does not work correctly, whereas the opposite is occurring, because the kidney is healthy even in a threatening situation. With current definitions of AKI, normalization of volaemia is needed before defining AKI in order to avoid this pitfall.

**SFAR Expert Coordinators**

Carole Ichai

**SRLF-Associated Expert Coordinators**

Christophe Vinsonneau

**Organizers**

Lionel Velly (SFAR), Bertrand Souweine (SRLF)

**SFAR Experts group**

Jean-Michel Constantin, Jacques Duranteau, Laurent Jacob, Olivier Joannes Boyau, Didier Journois, Matthieu Legrand, Laurent Muller, Thomas Rimmelé

**SRLF Experts group**

Emmanuel Canet, Christophe Clec’h, Michaël Darmon, Alexandre Lautrette, Nicolas Lerolle, Fabienne Tamion

**GFRUP Experts group**

Théophille Gaillot, Arnaud Garnier

**SFN Experts group**

Laurent Juillard, Eric Rondeau

**Composition of the working group**

**How to establish the diagnosis of acute kidney injury (AKI) and its severity**

A. Lautrette (Clermont-Ferrand), T. Rimmelé (Lyon), A. Garnier (Toulouse), T. Gaillot (Rennes)

**Strategies for the early diagnosis of AKI**

J. M. Constantin (Clermont-Ferrand), L. Jacob (Paris), M. Darmon, (Saint-Etienne), J. Duranteau (Paris), N. Lerolle (Angers)

**How to assess the risk of AKI**

C. Clec’h (Avicenne), M. Legrand (Paris)

**Strategies for the non-specific prevention of AKI**

M. Darmon (Saint-Etienne), L. Muller (Nimes)

**How to manage nephrotoxic agents**

M. Darmon (Saint-Étienne), O. Joannes-Boyau (Bordeaux)

**Strategies for the preventive and curative treatment of AKI**

E. Canet (Paris), D. Journois (Paris), A. Garnier (Toulouse), T. Gaillot (Rennes)

**Nutrition modalities during AKI**

F. Tamion (Rouen), B. Souweine (Clermont Ferrand), A. Garnier (Toulouse), T. Gaillot (Rennes)

**How to evaluate kidney function recovery after AKI**

E. Rondeau (Paris), C. Vinsonneau (Melun)

**Investigators in charge of references**

Fabien Armando (Nice), Yannick Walrave (Nice)

**Reading Group**

*Clinical Committee of Reporting Investigators* (*Sfar*): D. Fletcher, L. Velly, J. Amour, S. Ausset, G. Chanques, V. Compere, F. Espitalier, M. Garnier, E. Gayat, Jy Lefrant, Jm Malinovski, B. Rozec, B. Tavernier.

*Committee for Reporting and Evaluation* (*SRLF*): L. Donetti, M. Alves, Tboulain, Olivier B. Rissaud, V. Das, L. De Saint, Blanquat, M. Guillot, K. Kuteifan, C. Mathien, V. Peigne, F. Plouvier, D. Schnell, L. Vong.

*Board Meeting of Sfar*: C. Ecoffey, F. Bonnet, X Capdevila, H. Bouaziz, P. Albaladejo, L. Delaunay, M.-L. Cittanova Pansard, B. Al Nasser, C.-M. Arnaud, M. Beaussier, M. Chariot, J.-M. Constantin, M. Gentili, A. Delbos, J.-M. Dumeix, J.-P. Estebe, O. Langeron, L. Mercadal, J. Ripart, M. Samama, J.-C. Sleth, B. Tavernier, E. Viel, P. Zetlaoui.

*Board Meeting of SRLF*: P.-F. Laterre, J.-P. Mira, J. Pugin, X. Monnet, C.-E. Luyt, J.-L. Diehl, S. Dauger, J. Dellamonica, B. Levy, B. Megarbane, Pr Benoît Misset, H. Outin, F. Tamion, S. Valera.

## Background

Acute kidney injury (AKI) is a syndrome that has progressed a great deal over the last 20 years. The decrease in urine output and the increase in classical renal biomarkers, such as blood urea nitrogen (BUN) and serum creatinine (Scr), have largely been used as surrogate markers for decreased glomerular filtration rate (GFR), which defines AKI. However, using such markers of GFR as criteria for diagnosing AKI has several limits including the difficult diagnosis of non-organic AKI, also called “functional renal insufficiency” or “pre-renal insufficiency”. This situation is characterized by an oliguria and an increase in creatininemia as a consequence of a reduction in renal blood flow (RBF) related to systemic haemodynamic abnormalities. In this situation, “renal insufficiency” seems rather inappropriate as kidney function is not impaired. On the contrary, the kidney delivers an appropriate response aiming to recover optimal systemic physiological haemodynamic conditions. Considering the kidney as insufficient is erroneous because this suggests that it does not work correctly, whereas the opposite is occurring, because the kidney is healthy even in a threatening situation. With current definitions of AKI, normalization of volaemia is needed before defining AKI in order to avoid this pitfall.

In addition, numerous data highlight that Scr has strong limitations, which make it an imperfect surrogate marker for assessing GFR and consequently AKI.

However, because its use has long been standardized around the world and it is easy and inexpensive to measure, SCr remains the dominant renal biomarker used in the current definitions of AKI.

**Fig. 1:**
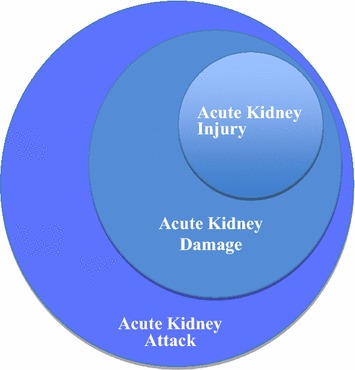
Acute kidney disease: from attack to dysfunction

The literal translation between related French and English terminologies can be confusing (Fig. 1)

1. *Acute kidney injury* (*AKI*) is diagnosed thanks to one clinical criterium (urine output) and one biomarker for renal function (SCr). Despite frequent situations in which renal parenchymal damage is generally present, this definition is focused on kidney “dysfunction” (in terms of the inability of the kidney to maintain homoeostasis due to a reduction in GFR). Thus, AKI has replaced the use of the older term “acute renal failure” (ARF), which corresponds to the most severe level of AKI and is characterized by clinically relevant renal failure.
2. *Acute kidney damage* (*AKD*) refers to renal parenchymal damage that may be evidenced via histological samples or by biomarkers of renal tissue damage but not by measures of renal function. Finally,
3. *Acute kidney attack* refers to situations at risk of kidney injury and kidney dysfunction. This latter situation is frequently observed in various conditions such as in sepsis, major surgery and nephrotoxic agent administration.

These different notions of AKI and damage have emerged over the last few years, partly due to the discovery of new biomarkers for renal function that allow clinicians to accurately assess kidney damage, and consequently renal dysfunction, before any subsequent change in the classical parameters of AKI.

Clinicians must know that kidney injury is not synonymous with renal failure and that AKD and attack develop as part of the continuum of AKI. These notions are essential since they allow clinicians to describe the conditions in which a therapeutic action might avoid or reduce the risk of worsening ARF. Growing experimental and clinical research actively seeks to assess the role of these renal biomarkers in detecting early AKI.

## Methods

The working method used to elaborate these recommendations is the GRADE^®^ method. Following a quantitative literature analysis, this method is used to separately determine the quality of available evidence on the one hand (i.e. a confidence estimation needed to analyse the effect of the quantitative intervention) and a level of recommendation on the other. The quality of evidence is distributed into four categories:

- High: further research is very unlikely to change the confidence in the estimate of the effect.
- Moderate: further research is likely to have an impact on the confidence in the estimate of the effect and may change the estimate of the effect itself.
- Low: further research is very likely to have an impact on the confidence in the estimate of the effect and is likely to change the estimate of the effect itself.
- Very low: any estimate of the effect is very unlikely.

The analysis of the quality of evidence is completed for every study; then, a global level of evidence is defined for a given question and criterion. The final formulation of recommendations will always be binary, positive or negative and strong or weak.

- Strong: we recommend or we recommend not to do (GRADE 1+ ou 1−).
- Weak: we suggest or we suggest not to do (GRADE 2+ ou 2−).

The strength of the recommendations is determined according to key factors and validated by the experts after a vote, using the Delphi and GRADE Grid method.

- The estimate of the effect.
- The global level of evidence: the higher the level of evidence, the stronger the recommendation.
- The balance between desirable and undesirable effects: the more favourable the balance, the stronger the recommendation.
- Values and preferences: in case of uncertainty or large variability, the level of evidence of the recommendation is probably weak, and values and preferences must be more clearly obtained from the affected persons (patient, physician and decision-maker).
- Cost: the greater the costs or the use of resources, the weaker the recommendation.
- The elaboration of a recommendation requires that 50 % of participants should have an opinion and that <20 % of participants prefer the opposite proposition.
- The elaboration of a strong recommendation requires the agreement of at least 70 % of participants.

The analysis of AKI management has been assessed according to seven themes: (1) AKI detection and diagnosis strategies; (2) AKI risk assessment; (3) non-specific AKI prevention strategies; (4) nephrotoxic agent management; (5) pharmacological strategies for the preventive and curative treatment of AKI; (6) AKI nutritional modalities; and (7) assessment of renal function recovery after AKI.

A specific analysis was performed for AKI in paediatric patients. A total of 24 experts were separated into nine working groups (the paediatric experts were involved in all questions).

Publications had to have taken place after 1999 to be selected. In case of an absence or a very low number of publications during the considered period, the timing of publications could be extended to 1990.

The level of evidence of the literature focused on AKI is globally associated with a weak level of methodology. The experts were, therefore, faced with three situations:

- For some questions, in the existence of several trials or meta-analyses with an acceptable methodological quality, the GRADE^®^ method was totally applicable and allowed recommendations.
- When no meta-analysis was available to answer the question, a qualitative analysis by the experts following the GRADE^®^ method was possible and a systematic review was performed.
- For some questions, in the absence of recent studies, no recommendation was possible.

After a synthesis of the experts, work and, implementation of the GRADE^®^ method, 33 recommendations were formally developed by the organizing committee. Among all recommendations, 9 were strong (Grade 1±) and 16 were weak (Grade 2±), and for eight questions, it was impossible to apply the GRADE^®^ method.

All of these recommendations were submitted to a reviewing group for a Delphi method assessment. After two rounds of voting and evaluation and after various amendments, a strong agreement was reached for 32 (99 %) recommendations.

## 1. How to establish the diagnosis of AKI and its severity

R1.1—We recommend to use the KDIGO criteria (stage 1) to define AKI based on the presence of at least one of these 3 following diagnostic criteria: (1) an increase in Scr ≥26.5 μmol/l within 48 h; (2) an increase in Scr ≥1.5-fold from baseline value within the last 7 days; and (3) urine output <0.5 ml/kg/h for 6 h.

(Expert opinion) STRONG Agreement

R1.2—We recommend to use the KDIGO classification to characterize the severity of AKI, according to the following table (Table 1).

**Table 1:** Classification of AKI according to the KDIGO criteria [1]

Stage	Serum creatinine	Urine output
1	≥26.5 μmol/l or 1.5–1.9 times baseline serum creatinine level	<0.5 ml/kg/h for 6–12 h
2	2.0–2.9 times baseline serum creatinine level	<0.5 ml/kg/h for ≥12 h
3	3.0 times baseline serum creatinine level ou serum creatinine ≥354 µmol/l or initiation of renal replacement therapy	<0.3 ml/kg/h for ≥24 h or anuria for ≥12 h

The stage is determined by the worse of either the “serum creatinine” or “urine output” criteria

(Expert opinion) STRONG Agreement

R1.3—To estimate GFR, we do not recommend the use of formulas (Cockroft-Gault, MDRD, CKD-EPI) in critically ill patients or in the post-operative period.

(Grade 1−) STRONG Agreement

R1.4—To estimate GFR, we suggest calculation of creatinine clearance using the following formula: measured creatinine clearance with the UV/P creatinine formula.

(Grade 2+) STRONG Agreement

**Rationale:** AKI is a clinical and biological syndrome with multiple causes and which includes various degrees of severity from AKD to ARF. The definition of AKI proposed in this recommendation is the international Kidney Disease Improving Global Outcomes (KDIGO) classification published in March 2012 [1]. As of 2015, there are no recent studies questioning this definition, and most of the available scientific trials that have focused on AKI and ARF use the KDIGO definition. AKI is defined by an increase in Scr which indicates a reduction in GFR. The best way to evaluate GFR is given by the calculated creatinine clearance using the formula UV/P (ml/min) (U being the urinary creatinine concentration in µmol/l, V the urinary volume expressed in ml per unit time, P the Scr concentration in µmol/l). This technique requires the collection of at least 1 h worth of urine (“flash creatinine clearance”) [2]. Estimated creatinine clearance formulas (sMDRD, CKD-EPI, Cockroft and Gault) must not be used because they were developed for stable patients with chronic renal insufficiency (CRI), but not validated in critically ill patients [2]. However, it is possible to use them carefully during pre-operative visits for surgical patients. An accurate assessment of GFR is essential for adapting the dosing of drugs, which are eliminated by kidney.

The KDIGO classification represents an optimized synthesis of both pre-existing classifications (RIFLE and AKIN), which were previously elaborated by an international expert group including nephrologists and intensivists aiming to characterize the severity of AKI [1, 3]. Since the publication of the RIFLE [4] and AKIN [5] classifications, a wide literature highlights that they are well correlated with AKI severity because the resulting morbidity (risk of requiring renal replacement therapy [6–9], in-ICU and in-hospital lengths of stay, risk of CRI development [10] and mortality rate increase proportionally with the stage of severity of these classifications [6–9]. Following AKI diagnosis, the determination of its severity is required for evaluating prognosis.

R1.1 Paediatrics—In paediatric patients, we suggest using the RIFLE classification modified for paediatric patients (pRIFLE) for diagnosing AKI: a decrease ≥25 % of estimated creatinine clearance or urine output <0.5 ml/kg/h during 8 h.

(Expert opinion) STRONG Agreement

R1.2 Paediatrics—In paediatric patients, we suggest evaluating the severity of AKI by using the criteria of the pRIFLE classification.

(Experts opinion) STRONG Agreement

**Rationale:** In 2012, KDIGO recommendations for defining AKI were formulated for both adult and paediatric patients. However, RIFLE and AKIN criteria are not really appropriate for children as they do not take into account the large variations in body mass index found in these patients. Moreover, because muscle mass is lower in children than in adults, Scr values are not a good marker of paediatric AKI. Consequently, a paediatric RIFLE classification (pRIFLE) based on an estimated Scr clearance and urine output has been proposed by Akcan-Arikan et al. [11]. In these modified criteria, AKI is defined by the presence of at least one of the following:

- a decrease in the estimated creatinine clearance >25 %
- a urine output <0.5 ml/kg/h for 8 h

In this classification, the estimated creatinine clearance is calculated according to the Schwartz’s paediatric formula [12] and is compared to the reference value of 100 ml/min/1.73 m^2^ if the previous value of the patient is unknown, which is frequent in this population. This pRIFLE classification has been subsequently validated in various prospective studies considering children in ICUs or those in the early post-operative period after cardiac surgery [13]. By extension, the severity of AKI is also based on the pRIFLE (Table 2).

**Table 2:** Diagnosis and severity criteria of AKI in paediatric patients

Grade	Estimated plasma creatinine clearance	Urine output
Risk	Decrease >25 %	<0.5 ml/kg/h during >8 h
Injury	Decrease >50 %	<0.5 ml/kg/h during >16 h
Failure	Decrease >75 % or <35 ml/min/1.73 m^2^	<0.3 ml/kg/h during 24 h or anuria >12 h
Loss	Grade «failure» persisting for >4 weeks	
End stage (chronic renal insufficiency)	Grade «failure» persisting for >3 months	

## 2. Strategies for the early diagnosis of AKI

R2.1—We recommend not to use renal biomarkers to diagnose early AKI.

(Grade 1−) WEAK agreement

**Rationale:** As mentioned in the introduction, regardless of its severity, AKI is characterized by renal dysfunction expressed by an increased Scr concentration or a decrease in urine output. When considering pathophysiology, this phase is always preceded by kidney attacks (of mostly haemodynamic or inflammatory nature), which can lead to irreversible parenchymal kidney damage and finally renal dysfunction when repeated [14, 15]. Currently, no curative strategies enable clinicians to treat such established damage, and AKI is clearly associated with an increased independent risk of in-hospital mortality and CRI within a few years following AKI [16–21]. Therefore, current data strongly suggest the need to research risk factors for AKI and to detect early kidney attack episodes [1, 22–24]. Consequently, over the last 10 years multiple renal biomarkers capable of detecting early acute kidney attacks have been developed. These biomarkers are essentially proteins synthesized subsequent to renal damage.

Numerous studies have evaluated the relevance of plasma and urinary renal biomarkers for diagnosing early AKI [1, 25, 26]. This evaluation, which is still ongoing for several of them, reports sensitivity ranging from 70 to 92 % and specificity from 70 to 95 %, depending on the nature of the biomarker, the sample type (plasma or urine) and, above all, the threshold level used to define AKI. Cystatin appears to be a biomarker for GFR and consequently an earlier and more efficient functional marker than Scr. A recent meta-analysis including 19 studies and 3336 patients reports that plasma cystatin has a sensitivity of 71 % and a specificity of 92 %, a predictive value which is higher than that of urinary cystatin and Scr [27]. Renal tubular biomarkers indicate kidney tissue damage. Among them, the most evaluated are kidney injury molecule-1 (KIM-1), neutrophil gelatinase-associated lipocalin (NGAL), lipid acid-binding protein (L-FABP), interleukin-18 (IL-18), β2-microglobulin and cell cycle arrest biomarkers [insulin growth factor-binding protein 7 (IGFBP 7) and tissue inhibitor of metalloproteinase-2 (TIMP-2)]. A recent meta-analysis including 23 studies and 4512 patients showed a moderate sensitivity and specificity for IL-18 measurements [28]. KIM-1, which has also been evaluated in a recent meta-analysis, seems to be an accurate biomarker for the diagnosis of early risk of AKI [29]. As found in a meta-analysis performed in 2009, urinary and plasma NGAL determination seems to be useful for the early diagnosis of AKI [30]. This study also demonstrated that NGAL has a good predictive value with reference to mortality rates and renal replacement requirements (RRTs) during ICU hospitalization. Similar to data related to other biomarkers, this meta-analysis confirms that NGAL measurements can be viewed as an accurate method for facilitating the early detection of kidney damage while no real benefit has been shown for the diagnosis of renal dysfunction. Recently, the same authors have reported similar results based on several prospective observational studies, leading to a cohort of 2322 critically ill patients [31]. However, results issued from this latter meta-analysis show a high heterogeneity between studies related to various conditions, abnormal cut-off values and measurement timings. Both IGFBP 7 and TIMP-2 biomarkers for cell cycle arrest have been assessed and compared with other major renal biomarkers in 738 critically ill patients at risk of AKI [32]. Results show that, in patients with various pathologies, a combined IGFBP7 and TIMP-2 measurement results in a higher sensitivity and specificity as compared with all other studied biomarkers (AUC = 0.8). Moreover, the risks of death and of RRT increase with high levels of these two biomarkers. A recent study with a cohort of 420 patients has confirmed that this biological tool is relevant for detecting and diagnosing early renal damage [33].

While data largely support that these biomarkers are useful for assessing early kidney damage and severity, the performance of these biomarkers and their daily use raise several problems. These various biomarkers indicate different mechanisms of injury: ischaemia, hypoxia, cellular regeneration or cell cycle arrest. Their syntheses are located in different sites, and they are activated with different kinetics following kidney injury [23, 26, 34]. Despite a growing literature, there is no study that truly demonstrates their utility in clinical practice for critically ill patients at risk of AKI. Several reasons preclude the implementation of such tools in current practice: multiple biomarkers, no real specificity, various kinetics of synthesis, impact of the pre-existing renal dysfunction, impact of the causal pathway leading to AKI and high costs. Therefore, until now, there is no ideal renal biomarker and the future use of these tools points towards combined and repeated measurements within time (kinetics). Finally, there are no data demonstrating the utility of such measurements for diagnosing AKI with dysfunction, which is simply based on Scr modifications or urine output, or for its therapeutic management.

In summary, there is at this time no randomized controlled study allowing experts to recommend the measurement of one or several renal biomarkers in order to diagnose AKI, which is already associated with renal dysfunction. On the other hand, the literature gives a strong signal that these biomarkers are useful tools that facilitate the early diagnosis of AKD, a stage which increases both the risks of AKI and death in critically ill patients. In the light of the current context, the generalization of such measurements remains difficult, especially as concerns the need to choose one or several biomarkers, the clinical relevance, significance thresholds and the timing for measurement(s), all of which are parameters that require more knowledge according to the type of patients.

Rationale for paediatric patients: It is not possible to directly extrapolate results issued from studies performed on adults to paediatric patients. Indeed, multiple parameters such as aetiology or treatment of AKI, presence of comorbidities and anthropometric characteristics are strongly different between paediatric and adult patients [35]. However, prospective trials performed in paediatric populations, especially in the post-operative cardiac surgery period, suggest that urinary renal biomarkers could be useful for the early diagnosis of AKI [36]. Moreover, data issued from a prospective study in critically ill paediatric patients have shown that estimated GFRs based on cystatin C levels have a higher sensitivity than those based on the usual equations using Scr [37].

R2.2—We suggest not to use the Doppler renal resistive index to diagnose or treat AKI.

(Grade 2−) STRONG agreement

**Rationale:** Measurement of renal velocity by Doppler ultrasound is a non-invasive and rapid surrogate method that allows the instantaneous assessment of parenchymal renal perfusion [38, 39]. Because of its easily accurate and repeatable measurements, this approach supposedly allows the assessment of modifications of RBF in response to therapeutic management. Therefore, the past 10 years have seen considerable growth in research on critically ill patients in order to evaluate the performance of Doppler ultrasound during AKI. Research has been developed in two ways.

The first research trend is focused on the use of the resistive index as a tool for measuring intrarenal haemodynamics. A recent experimental study reported that measuring RBF was impossible using Doppler sonography. Indeed, there was no relationship between measured RBF and estimated RBF by sonographic Doppler and the time for ultrasonographic transit, which was previously validated for regional blood flow measurements [40]. The same study highlighted that the interpretation of variations in the resistive index is difficult due to a very low relationship between variations in RBF after various therapeutic manoeuvres and variations in resistive index [40]. The most predictive parameter of 20 % variation in RBF issued from the Doppler was the variation in diastolic velocity, but the AUC was poor (0.75) [40]. Several authors have assessed resistive index variation in response to increasing doses of noradrenaline or “renal low” dose dopamine. Results suggest that the resistive index might be a guided goal measurement allowing the optimization of catecholamine doses in order to potentially improve renal perfusion [41, 42]. Finally, a further study did not find any variation in the resistive index following an intravascular load, regardless of pre-existing renal function and the response to this load as assessed by a variation in the systolic ejection volume [43]. Despite promising results, these preliminary studies show a poor level of proof. Both the significance of resistive index variation and doubts related to the reproduction of measurements are major limitations for the interpretation of these results as well as for their recommendation in daily practical clinical use.

The second renal Doppler ultrasound research trend is focused on the assessment of renal prognosis. An observational study including 37 patients in septic shock suggests that the resistive index might help predict the occurrence of AKI on day 5 [44]. Several other preliminary trials also suggest that the resistive index would allow clinicians to distinguish pre-renal (non-organic) from intrarenal (organic) AKI [45–47], to predict renal function outcome at days 3 or 5 [43, 48–50] and to predict RRT [51]. Thus, these studies suggest that the resistive index could be a tool for assessing renal prognosis with a good sensitivity and specificity [45, 47–51]. Certain studies performed on non-critically ill septic patients (35–91) have small sample sizes, which are mostly monocentric in nature or have poor methodological design [45–47, 49, 50]. A recent study including 94 patients reported contradictory results and suggested that the resistive index had a limited performance when used to evaluate renal prognosis [52]. This contradictory set of results could be explained by two limitations associated with this technique. The first is the significance of the resistive index values and variations, which remains unclear because the relationship between this parameter and renal vascular resistances seems to be poor, perhaps due to a large number of additional factors implicated in AKI [38, 39, 53–56]. Indeed, experiments performed on models of ex vivo kidneys have confirmed a major role for vascular compliance (modification in vascular diameter following changes in pressure) or pulse pressure as a determinant of the resistive index [54–56]. These experimental data have been recently confirmed in kidney-transplanted patients [57]. The second limitation is the feasibility and reproducibility of measures: a recent study confirms the feasibility of resistive index measurement after a short session of education for inexperienced intensivists, but the reproducibility between experienced and inexperienced ones is poor with variations in the resistive index reaching ±0.1 [50]. Taking into account the available data, this promising technique deserves to be further evaluated and cannot be recommended for current use.

## 3. How to assess the risk of AKI

R3.1—We recommend looking for risk factors for AKI related to the patient’s susceptibilities and/or to exposures (Table 3).

**Table 3:** Major risk factors for AKI related to patient susceptibilities and/or to exposures in hospitalized patients

Underlying susceptibilities	Procedures/exposures
Age ≥65 years^a^	Sepsis^a^
Chronic kidney disease^a^	Haemodynamic instability
Male	Perioperative period^a^
	Major surgery^a^ (emergency, abdominopelvic, cardiovascular, thoracic, bleeding surgeries)
African origin	Severe burn
Obesity (BMI >40 kg/m^2^)	Severe trauma
Arterial hypertension	Nephrotoxic agents (drugs, radiocontrast agents)
Congestive cardiac insufficiency	
Hepatocellular insufficiency	
Severe respiratory insufficiency	
Diabetes	
Cancer	
Anaemia	

^a^Most important risk factors

(Experts opinion) STRONG Agreement

**Rationale:** In hospitalized patients, all the different predictive scores for AKI reported in the literature describe more or less the same risk factors, which are related to patient susceptibilities and exposures. However, the weight of each of risk factors differs according to the type of patient and the surrounding procedures. The greater the number of risk factors, the greater the risk of AKI. The two most important risk factors related to patient susceptibilities are age and pre-existing chronic kidney disease. The age threshold above which the risk is present varies in the literature, from 55 to 65 years old, according to the context. Among procedure-related risk factors, the most frequently found are sepsis and surgery, or even better the perioperative period [15, 58, 59]. The aim of this chapter is neither to supply an exhaustive review of these predictive scores [15], nor to create a global score for all patients and all procedures. For example, it has been shown that prolonged extracorporeal circulation (over 2 h) is a major risk factor for AKI during cardiac surgery. Kheterpal et al. [59] defined a score (“AKI Risk Index”) designed to predict the development of AKI after non-cardiac major surgery. This score is based on the attribution of points for each of the nine risk factors as follows: age ≥56 years, male sex, congestive cardiac insufficiency, ascites, arterial hypertension, emergency surgeries, intraperitoneal surgery, CRI (pre-operative Scr ≥1.2 mg/dl) and diabetes (oral or insulin treatments). The ROC curve associated with this score was 0.80 ± 0.02. The incidence of AKI increased from 0.2 % in patients with 0–2 risk factors to 9.5 % in those with more than five risk factors, and this incidence increased with the number of risk factors.

Table 4 summarizes the major nephrotoxic agents that are most frequently used in ICUs and during the perioperative period [60, 61]. Among them, some could be replaced by non-nephrotoxic agents or administered using associated preventive measures (see the corresponding chapters).

**Table 4:** Major nephrotoxic agents responsible for AKI in ICUs and in the perioperative period

Radiocontrast agents
Aminoglycosides
Amphotericin
Non-steroidal anti-inflammatory agents
β-Lactams (interstitial nephropathies)
Sulfamides
Aciclovir, methotrexate, cisplatin
Cyclosporin, tacrolimus
Angiotensin-converting-enzyme inhibitors (ACE)

The identification of patient exposure to nephrotoxic agents/procedures is essential for correctly adjusting patient monitoring and management. In high-risk situations, the close monitoring of urine output and Scr in ICUs would help assess the evolution of renal function and the efficiency of strategies for preventing AKI (see corresponding chapters). If AKI occurs, the same type of measures will continue in order to limit a worsening of function and avoiding the further compromise of renal recovery.

R3.2—In high-risk situations, we suggest monitoring urine output and Scr to detect the development of AKI and apply the appropriate preventive measures.

(Experts opinion) STRONG agreement

## 4. Strategies for the non-specific prevention of AKI

R4.1—We recommend not administering hydroxyethyl starch (HES) in the ICU.

(Grade 1−) STRONG agreement

R4.2—We suggest the preferential use of crystalloids instead of colloids for fluid loading.

(Grade 2+) STRONG agreement

**Rationale:** Several randomized or observational studies and meta-analyses have examined the beneficial effect of a preferential administration of colloids compared to crystalloids [62–73]. In the ICU, the use of HES, regardless of its type, has been reported to be associated with an increase in mortality rate, AKI incidence and the need for RRT in several meta-analyses with a high level of proof [58–71, 73]. A Cochrane meta-analysis simultaneously considering all colloids did not find any beneficial effect associated with the preferential use of colloids, regardless of nature, compared with crystalloids. The same study reported an increased risk of death related to HES administration [72]. Only one recent randomized controlled study suggested a decrease in 90-day mortality (secondary endpoint) associated with fluid vascular loading performed with colloids, the absence of deleterious effects in terms of 28-day outcomes (primary endpoint) and the absence of a higher risk of AKI (secondary endpoint) [15]. However, in this study, judgement criteria in favour of colloids were secondary in nature and the weakness of the benefit led the authors to consider their results as investigational only [15]. Therefore, these results cannot justify preferential use.

A recent meta-analysis performed during the perioperative period, including several studies with low levels of proof, did not find any effect of fluid solution on AKI occurrence [74]. The low prevalence of AKI in this population and thus the low power of studies considered in this meta-analysis make it difficult to interpret this result [74].

In terms of fluid and sodium balance or haemodynamic stability, the benefit of colloids seems limited. Indeed, in two trials with a high level of proof for assessing efficiency of fluid balance and vascular load, a limited difference in favour of HES was found. In Myburgh’s et al. trial [63], the difference in terms of fluid balance on day 4 corresponded to 61 ml (982 ± 1069 vs 921 ± 1161, *p* = 0.03). Guidet et al. [67] found that the difference in fluid vascular load was 331 ml (1709 ± 1164 vs 1379 ± 886 ml, *p* = 0.02). However, in this latter study, fluid balance was similar between both groups on day 4 (56.6 vs 55.8 ml/kg).

On 11 October 11 2013, the European Agency for Medicines (EMA) made public its conclusions concerning the administration of HES [75]: HES must not be used in septic, critically ill or burned patients. Their administration remains possible in case of haemorrhage shock when crystalloids are not sufficient (for a period not exceeding 24 h, and in association with renal function monitoring for 90 days).

R4.3—We suggest preferring balanced solutions in case of large fluid vascular loading.

(Grade 2+) STRONG agreement

**Rationale:** At this time, there are no randomized studies demonstrating any beneficial effect in terms of mortality when solutions with a low chloride concentration are preferentially administered in critically ill patients or in the perioperative period. However, experimental data show that hyperchloraemia may cause renal vasoconstriction in a manner proportional to severity [76–78]. One clinical trial has found that an infusion of 2 l of 0.9 % saline is associated with a decrease in cortical renal perfusion (assessed by magnetic resonance imaging) as compared with an infusion of balanced solutions in healthy volunteers [79]. All data issued from observational cohort studies with or without propensity scores and matching on large samples highlight the deleterious effects of non-balanced solutions, especially on the kidney [80, 81]. Three recent, large, observational trials show that volume loading with 0.9 % NaCl is associated with increased morbidity, especially for kidney dysfunction, as compared with volume loading using balanced solutions [82–84]. In an observational study including 30,994 patients with abdominal surgery, Shaw et al. [82] found that patients receiving 0.9 % NaCl compared to those receiving balanced solutions had a higher rate of post-operative complications and RRT (4.8 vs 1 %, *p* < 0.05). Similar results have been found in the sequential observational study performed by Yunos et al. [83] in critically ill patients. In a cohort of 5000 surgical patients matched with a propensity score, McCluskey et al. [84] also found that hyperchloraemia was an independent risk factor for post-operative AKI. However, there is currently no real randomized controlled trial that confirms these data and results concerning mortality remain uncertain [82, 85]. A recent meta-analysis including more than 6000 patients concluded that the use of crystalloids rich in chloride increases the risk of AKI and blood transfusion, without affecting mortality rates as compared with balanced solutions [86]. Finally, considering these data, we consider non-balanced solutions as potentially deleterious, especially for kidneys, and suggest minimizing their use, especially for large-volume resuscitation [87].

R4.4—We recommend maintaining a minimal level of mean arterial pressure (MAP) between 60 and 70 mmHg to prevent and treat AKI.

(Grade 1+) STRONG agreement

R4.5—We suggest considering that patients with chronic arterial hypertension require a MAP target >70 mmHg.

(Grade 2+) STRONG agreement

**Rationale:** The optimal level of MAP during AKI has been rarely assessed. Because a MAP level of 65 mmHg is a survival factor in critically ill patients [88], especially during sepsis [89–91], this value is usually considered as the lowest acceptable level required for maintaining renal perfusion during AKI [92]. A diastolic arterial pressure <50–55 mmHg is associated with an increased AKI occurrence during septic shock [93, 94]. Because the threshold for renal autoregulation may be higher in the elderly and patients with a cardiovascular history, the requirement of maintaining MAP above a 65-mmHg threshold is frequently questioned for these patients. In terms of mortality, a recent multicentre randomized trial showed that a level of 80–85 mmHg had no beneficial effect compared with a level of 65–70 mmHg [95]. Two observational studies with a short follow-up (24 h) found no benefit for kidney function between a MAP of 65 versus 85 mmHg [96, 97]. However, several studies suggest that a MAP above 65 mmHg may be beneficial for the management of AKI [92, 95, 98]. In a randomized trial performed with 776 patients with septic shock, a MAP level of 80–85 mmHg (vs 65–70 mmHg) was associated with a decrease in the risk of RRT in patients with chronic arterial hypertension, while mortality rates were similar in both groups [95]. During AKI, a retrospective study with a cohort of 274 patients with sepsis suggested that a MAP <75 mmHg predicts the need for RRT [98]. In a retrospective trial including 423 patients, a level of MAP <75 mmHg was associated with an increase in AKI severity during septic shock [92]. These data underline the need to personalize the MAP threshold for each patient and to allow a MAP of 75–85 mmHg in patients suffering from persistent renal dysfunction despite an appropriate fluid load and a MAP of 65 mmHg.

In an observational study including 33,300 patients with non-cardiac surgery, a MAP <55–60 mmHg was associated with an increase in post-operative AKI [99]. In cardiac surgery, a MAP under 50 mmHg was associated with an increased rate of post-operative AKI, whereas a MAP of 60–70 mmHg was a protective factor [100]. In the same context, a 26-mmHg decrease in MAP was associated with a higher rate of AKI after cardiac surgery [101].

R4.6—We recommend monitoring and optimizing systolic ejection volume or derived parameters during the perioperative period in order to guide vascular fluid loading.

(Grade 1+) STRONG agreement

R4.7—We suggest applying the same recommendations in the ICU.

(Grade 2+) STRONG agreement

R4.8—After haemodynamic stabilization, we suggest avoiding fluid overload in the ICU.

Grade 2+) STRONG agreement

**Rationale:** In the perioperative period, regardless of the type of surgery, intraoperative haemodynamic optimization aiming at a cardiac index threshold of 4.5 l/min/m^2^, an oxygen delivery of 600 ml/min/m^2^ or an oxygen consumption of 170 ml/min/m^2^ allows clinicians to limit hypovolaemic episodes and consequently reduces the risk of post-operative AKI [102, 103]. These goals can be reached using a pulmonary arterial catheter, oesophageal Doppler or devices using arterial pulse contour analysis. The means include vascular fluid loading, vasopressor agents and inotropic drugs. Similar recommendations can be made in ICUs, but the level of proof is low.

In the ICU, fluid overload is associated with an increased incidence in AKI and its severity, regardless of the need for RRT [21, 104–108]. An increase in weight above 10 % is the most frequently studied parameter. All studies concerning this point are purely observational; comparative studies do not currently exist. Despite the relationship between fluid overload and AKI frequency and severity, there are no data, demonstrating that the control of fluid overload may have a beneficial renal effect. In other words, it has not been demonstrated that fluid overload is the cause or the consequence of AKI. Such fluid overload can be a simple marker of severity and not the cause of AKI. In the perioperative period, the control of fluid balance limits post-operative complications. During AKI, no benefit of controlling fluid balance has been demonstrated for kidney function [109, 110].

R4.9—We suggest using noradrenaline as a first-line treatment for maintaining MAP goals if a vasopressor drug is required.

(Grade 2+) STRONG agreement

**Rationale:** The use of vasopressors during AKI to reach or maintain the previously mentioned MAP and DAP goals is logical when fluid vascular load does not allow clinicians to reach them. Several observational studies show that noradrenaline is the vasoconstrictive agent of choice, which combines the best compromise in term of cost, safety and ease of use [111–117]. There is no controlled study concerning this specific point. Terlipressin may be an alternative to noradrenaline in the absence of coronary artery disease [118, 119]. Vasopressin has been used in rare cases, but the low sample size of these studies does not allow particular recommendations.

R4.10—We suggest not delaying any additional examinations or potentially nephrotoxic agent administration if they are needed to manage the patient.

(Experts opinion) STRONG agreement

## 5. How to manage nephrotoxic agents?

R5.1—We suggest optimizing hydration using crystalloids to prevent contrast-induced nephropathy (CIN), ideally before contrast media infusion and to continue this therapy within 6–12 h after this infusion.

(Grade 2+) STRONG agreement

R5.2—We suggest not using *N*-acetylcysteine (NAC) and/or sodium bicarbonate to prevent CIN.

(Grade 2−) STRONG agreement

**Rationale:** Despite a small number of studies with poor methodology, using numerous different definitions, the incidence of CIN in non-ICUs varies from 2 % in patients without any risk factor [120] to 25 % in those with risk factors (chronic kidney disease, diabetes, concomitant administration of nephrotoxic agents) [121]. In the ICU, according to the definition used, this incidence varies from 16 to 31 % [122–125]. Several other risk factors for kidney injury are usually concomitantly found in critically ill patients (hypotension, sepsis, nephrotoxic drugs), making it difficult to consider contrast media directly and solely responsible for AKI. Thus, most studies evaluate post-operative cardiac surgery patients or cardiology patients, but there are few trials available in critically ill patients.

The available studies considered in a large number of meta-analyses, which are unfortunately heterogeneous and for the most part poorly conducted (especially as concerns older studies), lead to conflicting results [126–131]. Moreover, in more recent meta-analyses positive results favouring NAC are demonstrated only when they include published trials. When considering all studies, especially unpublished ones, this favourable result disappears [128, 132]. Alkalinization with sodium bicarbonate has been proposed for the prevention of CIN. Two recent meta-analyses have found a beneficial effect associated with sodium bicarbonate in terms of a decrease in AKI incidence, whereas there was no effect on the need for RRT and in-hospital mortality [130, 133]. Two recent prospective randomized trials did not confirm these later results. The first one demonstrated that hydration with 0.9 % saline was associated with a lower decrease in GFR and a lower incidence of CIN as compared with sodium bicarbonate (1 vs 9 %, *p* = 0.02) [134]. In the second study, the results found that there was no difference in the incidence of CIN between 0.9 % saline and sodium bicarbonate (3 vs 5.1 %, *p* = 0.23) [135]. Currently, though sodium bicarbonate may be a satisfactory alternative to 0.9 % saline, there is still no proof of any advantage associated with such a preventive strategy. Other drugs that have been assessed have never shown any undisputable positive effect and cannot be recommended [136, 137]. Finally, fluid vascular loading seems to be the most efficient prophylactic management [138, 139]. Such a strategy minimizes risk exposure when considering that a limited volume of fluids is needed in this indication (about 1000 to 1500 ml within several hours) and will not have deleterious consequences, except for patients with cardiac insufficiency or fluid overload. Moreover, the expected benefit in terms of decreased incidence of AKI following the procedure should maintain sufficient urine output to allow rapid body water elimination.

Finally, fluid vascular loading before procedures seems to be the most efficient preventive treatment for CIN with a largely positive benefit/risk ratio, provided one is careful in patients at high risk of decompensation following moderate fluid infusion.

R5.3—We suggest administering aminoglycosides when necessary with respect to the following rules:

- administer them with single dosing per day,
- monitor their residual level in case of more than a single infusion,
- administer them for a maximum of 3 days whenever possible.

(Grade 2+) STRONG agreement

R5.4—We suggest not using non-steroidal anti-inflammatory drugs (NSAIs), converting enzyme inhibitors (CEIs), and angiotensin 2 receptor antagonists in patients at risk of AKI.

(Experts opinion) STRONG agreement

**Rationale:** Studies showing renal and ear toxicity associated with aminoglycosides are old and based on a design of twice-daily administration without any consideration of residual serum concentration levels [140–142]. More recent studies have shown that toxicity was essentially related to high levels of residual serum concentration of the drug (more than 20 h after infusion), while the peak concentration (measured ½ h after infusion) was the parameter responsible for efficiency [143–150]. Current recommendations are based on these later trials, favouring a high peak serum concentration using high doses of aminoglycoside as boluses and close monitoring of residual serum concentrations to avoid renal toxicity. In practice, the determination of peak serum levels performed 30 min after the infusion of aminoglycosides should be used, and then, the following dose should be adapted in order to reach the recommended threshold. For the problem of toxicity, if the aminoglycoside is used for several consecutive days, the evaluation of the residual serum level 24 h after its administration should be performed before infusing a new dose, provided the serum concentration is below the recommended threshold. Moreover, it has been shown that prolonged exposure increases the occurrence of AKI, explaining the reason for limiting the duration of treatment to 3 days. Such a strategy allows clinicians to be efficient during the acute phase of sepsis while limiting toxicity [144]. This limitation does not apply to endovascular and osteoarticular infections with material, as well as endocarditis. In these latter situations, the prolonged administration of aminoglycosides over several days or weeks may be needed. Nevertheless, there is no study assessing the recommended usual strategy in these conditions. This recommendation is an expert opinion issued from results based on older studies evaluating toxicity.

Despite the absence of randomized controlled studies evaluating the cumulative use of nephrotoxic agents, studies with cohorts of patients and those evaluating toxicity show that the association of several nephrotoxic risk factors, especially the accumulation of several nephrotoxic drugs, exponentially increases the risk of AKI [60, 61]. This should be taken into account when choosing drugs for patients at risk of AKI by considering their indications and favouring essential drugs. For example, non-steroidal anti-inflammatory agents should be avoided in patients treated with both aminoglycosides and glycopeptides. Only essential drugs and alternative strategies should be favoured for decreasing nephrotoxicity [151–153].

## 6. Pharmacological strategies for the preventive and curative treatment of AKI

R6.1—We recommend not using diuretics in order to prevent or treat AKI; we suggest using them for treating fluid overload.

(Grade 1−) STRONG agreement

**Rationale:** AKI is a frequent organ failure in the ICU and during the perioperative period surrounding cardiovascular surgery and is associated with a high risk of morbidity and mortality. In two recent meta-analyses [154, 155], the administration of diuretics did not reduce the incidence and severity of AKI. For this indication, furosemide did not demonstrate any benefit in terms of in-hospital mortality, the need for and the number of sessions of RRT. Because AKI with fluid overload is associated with higher mortality rates [104, 107], diuretics can be proposed for treating fluid overload.

R6.2—We suggest not using sodium bicarbonate to prevent or treat AKI

(Grade 2−) STRONG agreement

**Rationale:** The prevention of AKI using sodium bicarbonate has been performed in a randomized controlled multicentre study (sodium bicarbonate vs 0.9 % sodium chloride) with a primary endpoint being the occurrence of AKI in the post-operative period [156]. The results found a higher incidence of AKI in the sodium bicarbonate group (83/174 [47.7 %]) compared with the control group (64/176 [36.4 %], odds ratio [OR] 1.60 [95 % CI 1.04–2.45], *p* = 0.03). This study was stopped early because there was an increased mortality in patients receiving sodium bicarbonate (11/174 [6.3 %] vs 3/176 [1.7 %], OR 3.89 [1.07–14.2], *p* = 0.03). Two other recent studies performed in cardiac surgery did not find any beneficial effect of sodium bicarbonate for preventing AKI [157, 158].

Concerning the treatment of AKI, a recent meta-analysis has assessed the administration of sodium bicarbonate for this indication [159]. The primary endpoint was in-hospital mortality; secondary endpoints were the need for RRT, renal recovery and global survival. Four studies were considered in this analysis, but none met the pre-defined criteria. Consequently, taking into account data issued from current literature, we do not recommend the administration of sodium bicarbonate to prevent or treat AKI.

In the special condition of rhabdomyolysis-related AKI, fluid vascular loading requirements seem to be established [160–162], but the nature of the fluid remains in discussion. Theoretical benefits for using sodium bicarbonate are described (inhibition of intrarenal vasoconstriction, inhibition of lipid peroxidation and decreases in myoglobin crystallization with Tamm–Horsfall protein). Nevertheless, for methodological reasons (small size, multiple interventions, non-randomized studies), the rare availability of such studies cannot be used to confirm that sodium bicarbonate is superior to other solutions used for vascular loading [163–167].

R6.3—We recommend not using the following treatments to prevent or treat AKI: mannitol, dopamine, fenoldopam, atrial natriuretic factor, NAC, insulin-like growth factor-1 (IGF-1), erythropoietin, adenosine receptor antagonists.

(Grade 1−) STRONG agreement

**Rationale:** Mannitol has been proposed as a preventive treatment of AKI during the perioperative period in traumatic brain injury, in patients with rhabdomyolysis or those undergoing coronarography. In most studies, it increases urine output while increasing or not decreasing the incidence of AKI [168–170].

Low-dose dopamine (1–3 µg/kg/min) induces renal vasodilation and natriuresis in healthy adults. This agent has been evaluated as a preventive treatment for AKI in multiple clinical situations: critically ill patients with SIRS, the perioperative period for aortic surgery, the post-operative period following liver or kidney transplantation. Most studies, which include a randomized, controlled multicentre trial, a meta-analysis and a systematic review, conclude that dopamine has no beneficial effect either in preventive or in the curative treatment of AKI [171–173].

Fenoldopam is a dopamine-1 receptor agonist without α or β adrenergic systemic effects. Seven studies have included 1218 patients and did not find beneficial effects associated with the preventive or curative administration of fenoldopam as concerns mortality and the need for RRT [174–180]. The benefit of fenoldopam in terms of AKI occurrence is difficult to evaluate due to heterogenous delays in administration and diagnosis criteria among studies. Four meta-analyses are available but show conflicting results: two of them reported a beneficial effect with a reduction in the risk of AKI, one did not find any beneficial effect and the last one did not analyse the risk of AKI [181–184]. Considering current data in the literature, it is recommended not to use fenoldopam for the prevention or treatment of AKI.

Several natriuretic peptides that can increase GFR have been proposed as preventive and curative therapies for AKI. Most of the prospective randomized controlled trials and 3 meta-analyses have not found any benefit associated with these agents [185–188].

The preventive or curative administration of NAC for AKI has not demonstrated any beneficial effect concerning the need for RRT and reductions in mortality [129, 189–192]. Apart for preventing CIN, studies were performed essentially in the perioperative period of cardiovascular surgery.

Based on the available studies, no beneficial effect associated with the preventive and curative administration of IGF-1 has been demonstrated for AKI [193, 194]. Currently, there are not enough data to recommend erythropoietin for preventing or treating AKI [195]. Only one study including 171 patients undergoing cardiac surgery (coronary artery bypass graft) has shown that erythropoietin (300 U/kg) administered before cardiac surgery for coronary artery bypass grafts, enabled a reduction in the risk of post-operative AKI compared with 0.9 % saline solution (8 vs 29 %, *p* = 0.03) [196].

In two pilot studies, rolofylline (an antagonist of A 1 adenosine receptors) versus placebo was administered in patients with acute cardiac insufficiency [197, 198]. Both found that rolofylline increased urine output and improved creatinine clearance (vs placebo). The largest and more recent prospective multicentre trial (*n* = 2033 patients) comparing rolofylline versus placebo in patients with acute cardiac insufficiency did not find beneficial effect of rolofylline in terms of survival, cardiac and renal function [199].

There are two special conditions requiring the preventive treatment of AKI: (1) high doses of methotrexate infusion [200]; (2) patients with a high risk of tumour lysis syndrome. Methotrexate at high doses (1–12 g/m^2^) is a treatment required for numerous malignant tumours. This drug can induce AKI (up to 40 % of patients in a recent study), which is due to direct tubular toxicity and intratubular precipitation [201]. Intravenous hydration (≥2 l/m^2^) and urine alkalinization are both recommended strategies for preventing AKI. Several historical observational and interventional studies highlighted that these strategies were associated with an increased methotrexate clearance and decreased episodes of severe toxicity [202–205]. Urine alkalinization decreases methotrexate crystallization [202]. Thus, it is recommended to alkalinize the urine of patients who must receive high doses of methotrexate (1–12 g/m^2^) in order to prevent AKI. About one-third of patients with a high risk of tumour lysis syndrome develop AKI [206, 207]. In this clinical condition, hyperuricaemia is one of the factors which contribute to AKI by different mechanisms [208]. In two randomized controlled studies and 2 recent meta-analyses, rasburicase administration was associated with a more rapid and profound decrease in uricaemia as compared with allopurinol [209–211]. However, there is no proof that rasburicase is associated with reduced AKI incidence [212]. Hyperuricaemia is only one of the numerous mechanisms implicated in renal injury during tumour lysis syndrome (phosphate and calcium crystals, hypovolaemia, renal tumour infiltration, nephrotoxic exposure, inflammation). In patients with a high risk of tumour lysis syndrome, rasburicase administration is proposed by four recent expert recommendations [198, 213–215].

## 7. Nutritional modalities for AKI

R7.1—We suggest following the same nutritional strategy rules in critically ill patients whether or not they have AKI (without renal replacement therapy).

(Grade 2+) STRONG agreement

R7.2—We recommend not limiting nutritional support in order to only prevent fluid overload and/or the need for renal replacement therapy.

(Grade 1−) STRONG agreement

**Rationale:** AKI has an impact on fluid balance and acid–base equilibrium, but also interferes with the metabolism of each macronutrient, generally towards hypercatabolism. Thus, the consequences of AKI on nutrition add to those related to the underlying pathology [216]. In patients with AKI, undernutrition is significantly associated with a high incidence of infectious complications, prolonged in-hospital length of stay and mortality [18]. Nutritional evaluation is complex because normally available markers (body mass index, impedancemetry) become inaccurate due to modifications in hydration status [217]. Nutritional support in injured patients with or without AKI must be similar, aiming at an appropriate energy and protein intake, muscle mass preservation, improvement in immune function and reduction in mortality [218]. Nutritional requirements must take into account hypercatabolism related to illness and the presence or not of RRT and its technique. These parameters, more than AKI itself, have a major impact on nutritional strategies. Indirect calorimetry remains the reference tool required to define patient energy requirements. When this device cannot be used, it is recommended to provide an energy target of 20–30 kcal/kg/day and a protein target of 1.5 g/kg/day, in the absence of RRT [219]. In case of RRT, an increase in protein supply including glutamine and micronutrients (vitamins and trace elements) is suggested [220]. Water-soluble vitamins from the B group (especially B1 vitamins and folates) are significantly eliminated during RRT [221].

R7.1 Paediatric—We suggest adapting protein intake according to the age of children with AKI.

(Grade 2+) STRONG agreement

**Paediatric rationale:** KDIGO recommendations in 2012 [1] have elaborated recommendations for the nutrition of paediatric patients with AKI that can be followed. The authors insist on the essential point for paediatric patients, i.e. the dynamics of growth and weight gain, which justify a higher nutrition intake than in adults. The KDIGO recommendations have proposed the following protein intake depending on the age of children presenting with AKI:

- 2–3 g/kg/day from 0 to 2 years,
- 1.5–2 g/kg/day from 2 to 13 years,
- 1.5 g/kg/day above 13 years.

## 8. How to evaluate kidney functional recovery after AKI

R8.1—We recommend considering patients with AKI as patients at high risk of developing CRI.

(Grade 1+) STRONG agreement

R8.2—We suggest assessing renal function in patients who presented AKI 6 months after the acute episode.

(Grade 2+) STRONG agreement

R8.3—We suggest defining the absence of renal functional recovery following AKI as follows: an increase in Scr above 25 % of its basal value or RRT dependancy.

(Grade 2+) STRONG agreement

**Rationale:** Severe AKI can be associated with a total or partial absence of renal functional recovery, leading to CRI. Paediatric studies using long-term follow-up were the first to show that patients considered as completely recovered based on biological data can progress towards CRI within the 3 following years in 10 % of cases [222]. An incomplete recovery can lead to a normalization of the usual biological parameters (Scr), despite a decreased number of nephrons. This phenomenon leads to higher renal susceptibility in case of a new injury or during physiological ageing.

 A recent review estimates the incidence of CRI after an acute injury at 25.8/100 patient-years and the incidence of end-stage kidney disease at 6.6/100 patient-years [10]. Thus, it is clear that there is a relationship between AKI and CRI. Moreover, several studies report that there is also a relationship between the severity of AKI and the increased risk of chronic damage with a twofold increase in end-stage CRI requiring dialysis at 10 years [223]. This evolution towards CRI is associated with an increased mortality rate. The study of Pannu et al. [224] found that as soon as renal function recovery remains below 125 % of pre-injured Scr, a nephrologic follow-up is required in order to detect long-term poor renal functional recovery. A study assessed 3877 patients and, among them, 1153 were followed for 3 months by a nephrologist. This study found that these patients had a significantly higher survival compared with the matched control group (RR 0.76, 95 % CI 0.62–0.93) [225]. Therefore, a systematic follow-up by nephrologists is advised in patients who present with AKI, regardless of their early renal function recovery.
